# Block Time: A Multispecialty Systematic Review of Efficacy and Safety of Ultrasound-guided Upper Extremity Nerve Blocks

**DOI:** 10.5811/westjem.56058

**Published:** 2023-06-30

**Authors:** Campbell Belisle Haley, Andrew R. Beauchesne, John Christian Fox, Ariana M. Nelson

**Affiliations:** * University of California, Irvine Medical Center, Department of Emergency Medicine, Orange, California; † Beth Israel Deaconess Medical Center, Department of Radiology, Boston, Massachusetts; ‡ University of California, Irvine Medical Center, Department of Anesthesiology, Orange, California

## Abstract

**Introduction:**

Ultrasound-guided peripheral nerve blockade is a common pain management strategy to decrease perioperative pain and opioid/general anesthetic use. In this article our goal was to systematically review publications supporting upper extremity nerve blocks distal to the brachial plexus. We assessed the efficacy and safety of median, ulnar, radial, suprascapular, and axillary nerve blocks by reviewing previous studies.

**Methods:**

We searched MEDLINE and Embase databases to capture studies investigating these nerve blocks across all specialties. We screened titles and abstracts according to agreed-upon inclusion/exclusion criteria. We then conducted a hand search of references to identify studies not found in the initial search strategy.

**Results:**

We included 20 studies with 1,273 enrolled patients in qualitative analysis. Both anesthesiology (12, 60%) and emergency medicine (5, 25%) specialties have evidence of safe and effective use of radial, ulnar, median, suprascapular, and axillary blocks for numerous clinical applications. Recently, multiple randomized controlled trials show suprascapular nerve blocks may result in lower pain scores in patients with shoulder dislocations and rotator cuff injuries, as well as in patients undergoing anesthesia for shoulder surgery.

**Conclusion:**

Distal upper extremity nerve blocks under ultrasound guidance may be safe, practical strategies for both acute and chronic pain in perioperative, emergent, and outpatient settings. These blocks provide accessible, opioid-sparing pain management, and their use across multiple specialties may be expanded with increased procedural education of trainees.

## INTRODUCTION

Over three million upper extremity traumas, 38.4% of which are digital injuries, present to emergency departments (ED) in the United States annually.^1^ Many of these injuries are treated with oral or intravenous analgesia, while more complicated cases often result in consultation with acute pain service. However, there is evidence that emergency physicians (EP) can provide safe and efficacious pain relief using ultrasound (US)-guided (upper extremity nerve blocks.^2^,^3^ Upper extremity nerve blocks are also used in outpatient settings by sports medicine, physical medicine and rehabilitation, and neurology.^4^–^6^ Despite this, most upper extremity nerve blocks are performed by anesthesiology perioperatively. It is less common for EPs to be trained in distal upper extremity blocks such as suprascapular, median, ulnar, and radial nerve blocks.^7^,^8^ Our goal in this review was to assess the current state of evidence behind US-guided upper extremity nerve blocks across all specialties.

Despite their widespread use in different specialties, there are no prior multidisciplinary systematic reviews of these blocks.^7^,^9^–^11^ In contrast, there are many published systematic reviews of brachial plexus blocks, which are standard of care for most upper extremity surgeries.^12^,^13^ Proximal brachial plexus blocks are rarely performed by EPs due to the perceived risk of pneumothorax and diaphragmatic paralysis, while distal blocks require skills already familiar to EPs, such as US-guided vascular access, fascia iliacus nerve block, and musculoskeletal ultrasound.^14^–^17^ These ultrasound applications are currently standard of practice for EPs.^18^ Nerve blockade has the potential to provide safe, opioid-sparing analgesia in the ED in vulnerable populations such as elderly patients, those suffering from substance use disorders, and children. Additionally, a recent survey of experts in emergency medicine (EM) supported the notion that US-guided regional anesthesia should be a developed curriculum in all EM residencies.^19^

Our objective in this review was to systematically evaluate the evidence supporting US-guided distal upper extremity nerve blocks across all specialties and determine their efficacy and safety. We hope to inform future educational initiatives in EM and encourage EPs to gain competence in US-guided nerve blocks to treat pain optimally.^20^

## METHODS

We searched MEDLINE and Embase databases to capture studies from the inception of each database to March 7, 2021. We developed a search strategy with guidance from sentinel articles using keywords from these articles as MeSH terms and consulted a medical librarian for a review of our search strategy (Appendix 1). Duplicate references were removed using EndNote. Titles and abstracts were screened by two independent investigators using the Rayyan screening app (Rayyan Systems Inc, Cambridge, MA) according to specific inclusions and exclusion criteria (Appendix 2).^21^ We included randomized controlled trials, case-controlled studies, cross-sectional studies, and cohort studies assessing the efficacy of US-guided upper extremity nerve blocks compared to other forms of analgesia or techniques of nerve block (Appendix 1).

Outcomes included those studies related to pain and safety. Studies taking place perioperatively, emergent settings, and outpatient clinical environments were all considered. Two authors (CBH and AB) were responsible for screening articles based on inclusion criteria, using the title, abstract and, if needed, the full-text article. Disagreements in article inclusion (6/85 studies) were reconciled through consensus development by further discussion of methods and outcomes using the defined inclusion/exclusion criteria. A hand search was also conducted by using the references of all full-text screened studies to identify studies that may have been missed from the search strategy. Our study was reviewed by local institutional review board (IRB), which determined this does not constitute human subject research and was, therefore, exempt from IRB review.

We created standardized forms to extract individual study data regarding study details and design, population characteristics, and results for outcomes of interest. We also completed risk of bias on all included studies using the revised Cochrane risk-of-bias tool (RoB 2).^22^

## RESULTS

**Figure 1:**
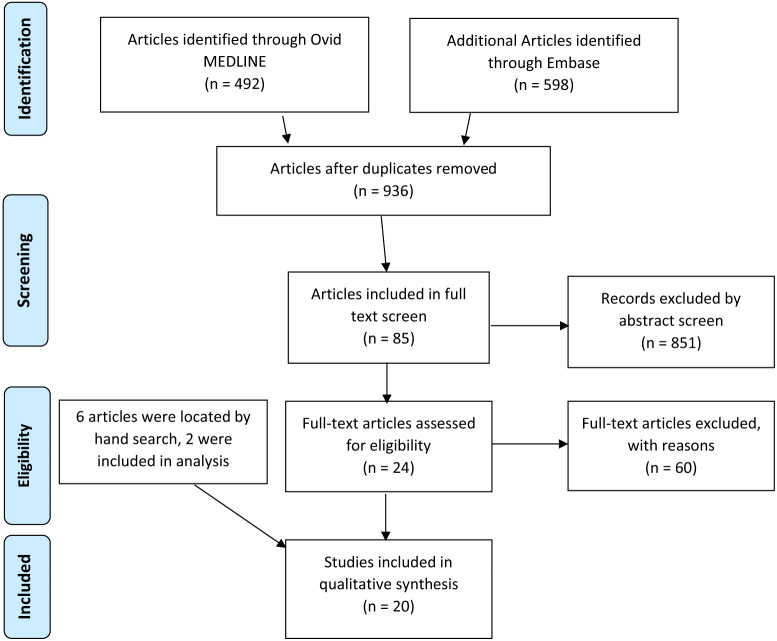
Flow diagram of search strategy for US-guided upper extremity blocks using PRISMA protocol. *US*, ultrasound; *PRISMA*, Preferred Reporting Items for Systematic Reviews and Meta-Analysis.

Our literature search and study selection process identified 936 abstracts by search of MEDLINE and Embase databases (Figure 1). Abstract screening identified a total of 85 references for full-text screening. Hand search of references in all full-text screened articles identified two additional studies for inclusion. A total of 20 studies were deemed eligible for inclusion (Appendix 2). These included studies from the specialties of anesthesia (60%), EM (25%), orthopedics (measured with multiple outcomes including reduction in scores on the visual analog scale(VAS), comparison to landmark-guided blocks, and comparison to pharmacologic pain control (Table 1). Studies that had overall positive outcomes by these measures were considered “effective” blocks for the purposes of this systematic review. Of the 20 total included studies, four studied block effectiveness for chronic pain.

### Mixed Forearm Blocks

Five publications studied the efficacy of combined forearm nerve blocks (median, ulnar, and radial nerves) (Table 1A). One randomized controlled trial (RCT) indicated median/radial/ulnar combined block was successful as primary anesthesia for hand surgery 97% of the time, while two single-arm, interventional studies in the ED indicated the effectiveness of combined blocks for hand injuries requiring procedural interventions.^7^,^9^,^10^ A retrospective cohort study analyzing all forearm blocks completed by the anesthesiology department at an academic center (536) showed no neurologic complications.^23^ One RCT of combined medial/ulnar blocks in patients undergoing carpal tunnel release surgery showed 93% effectiveness (Table 1B).^2^

### Individual Forearm Blocks

Two studies assessed the effectiveness of US-guided ulnar nerve block and showed a 100% success rate in blocking pinprick sensation (Table 1C).^24^,^25^ Three studies assessed median nerve block (Table 1D).^26^–^28^ One determined there was no difference in success when using hydrodissection with a median nerve block and reported 100% success of US-guided block of index finger/thenar eminence sensation.^26^ Another stated circumferential spread of the nerve block visualized on US increased success from 81–100% (*P* < 0.05).^28^ Finally, a study of pediatric patients undergoing trigger thumb surgery found 50 (100%) successful median nerve blocks in the US-guided group vs 50 (64%) using landmarks alone.^27^ Two prospective studies of radial nerve blocks demonstrated improvements in pain scores. In one, patients with Colles fracture undergoing reduction had VAS decrease from 8.2 (7.6–8.8) to 3.53 (2.7–4.3) with US-guided radial nerve block.^29^ The second study demonstrated improvement in hand osteoarthritis pain over 2–4 weeks.^30^

### Axillary and Suprascapular Shoulder Blocks

There were two RCTs of combined suprascapular and axillary blocks in the perioperative setting. One showed that in rotator cuff surgery the combination block was superior to suprascapular block alone, and the other found these blocks provided significantly less analgesia than the interscalene brachial plexus block for arthroscopic shoulder surgery (Table 2A).^31^,^32^

Five RCTs assessed the US-guided suprascapular nerve block (Table 2B). One ED study found this block decreased pain of shoulder dislocation reduction from mean VAS 85 (70–98) to 45 (33–45) and decreased time to discharge compared to sedation analgesia.^33^ Two studies in orthopedics studied the US-guided suprascapular block. One demonstrated improvement in Constant-Murley scores 12 weeks post-block for patients with rotator cuff tears compared to patients receiving subacromial injection; another found a nonsignificant change in VAS for patients with adhesive capsulitis.^34^,^35^ In one anesthesia study 18 of 83 patients (21.7%) experienced hemidiaphragmatic paralysis with US-guided suprascapular nerve block, while another found suprascapular blocks have less respiratory effect than brachial plexus blocks.^36^,^37^

### Risk-of-bias Assessment

We assessed RoB for 17 outcomes in 16 studies, including two cross-over studies (Appendix 4). Briefly, 14 outcomes (82%) had low RoB due to the randomization process, and 12 outcomes (71%) had low RoB due to deviations from the intended interventions. Additionally, all assessed outcomes (100%) had low RoB due to missing outcome data, and 13 (76%) had low RoB due to measurement of the outcome. Notably, 12 outcomes (71%) had some RoB due to the selection of the reported results, with the other studies having low RoB.

## DISCUSSION

**Figure 3:**
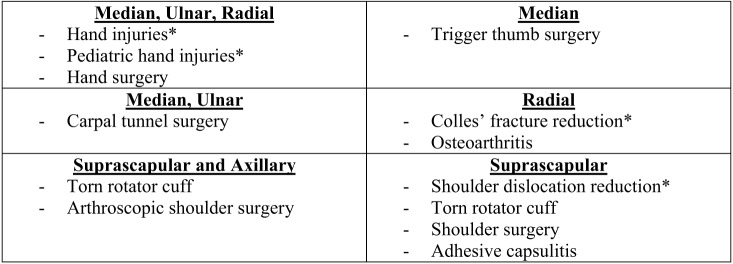
Clinical applications of ultrasound-guided distal upper extremities found on systematic review. *Study performed in the emergency department.

We evaluated the multispecialty evidence behind US-guided upper extremity nerve blocks to support future educational interventions in EM. Our review supports using upper extremity blocks for multiple indications in the ED (Figure 3).^7^,^23^,^32^,^36^ More broadly, this suggests high efficacy and few complications when performing upper extremity peripheral nerve blocks perioperatively and in the ED. Despite this evidence, there are no specific recommendations from the Accreditation Council for Graduate Medical Education or the American College of Emergency Physicians about which nerve blocks would be beneficial to patients presenting to EDs.^18^,^20^ However, a recent survey found significant support for an US-guided regional anesthesia curriculum for EM residents.^19^

Our findings also predict a benefit from more standardized EP education in US-guided regional anesthesia, which has the potential to decrease the need for procedural sedation and opioid use in the ED while providing safe, adequate analgesia for hand and forearm injuries.^36^ Populations such as patients with substance use disorder and older patients may benefit from regional pain management rather than central nervous system-active pharmaceuticals.

## LIMITATIONS

This study has several limitations, including our inability to make inferences between the specialties of anesthesia and EM. Regional anesthesia is a year-long fellowship in anesthesia, and anesthesia residents have formal training in regional nerve blocks throughout their residency. In many EDs, US-guided regional anesthesia has become standard, but this remains institution-dependent. Since we are comparing heterogeneous data (in terms of training and procedural skill), our study is limited to a systematic review rather than a meta-analysis.

To make truly quantitative statements, we would need the statistical analysis that only a meta-analysis would provide. This factor also limited our ability to fully follow PRISMA checklist guidelines. We also defined effectiveness broadly as a positive study result, but this specific was quite varied between the studies. Despite this, the finding that these procedures are low risk was consistent throughout our review of studies. Further research should be multifaceted and interventional. The most effective approach may be a multicenter RCT that includes both an educational initiative on US-guided nerve blocks for EM residents and a study determining effectiveness of these blocks compared to pharmacologic pain control.

## CONCLUSION

There is evidence that ultrasound-guided upper extremity nerve blocks are safe and effective based on multiple positive outcomes from different specialties. Improved training in US-guided nerve blocks in emergency medicine has the potential to provide a safe alternative to pharmacologic pain management or procedural sedation. In addition, given the significant intersection between the fields of anesthesia and EM in US-guided procedures, more formal educational collaboration may improve the technique and training of trainees by combining the considerable talents of both fields in performing these procedures.

## Supplementary Information





## Figures and Tables

**Figure 2:**
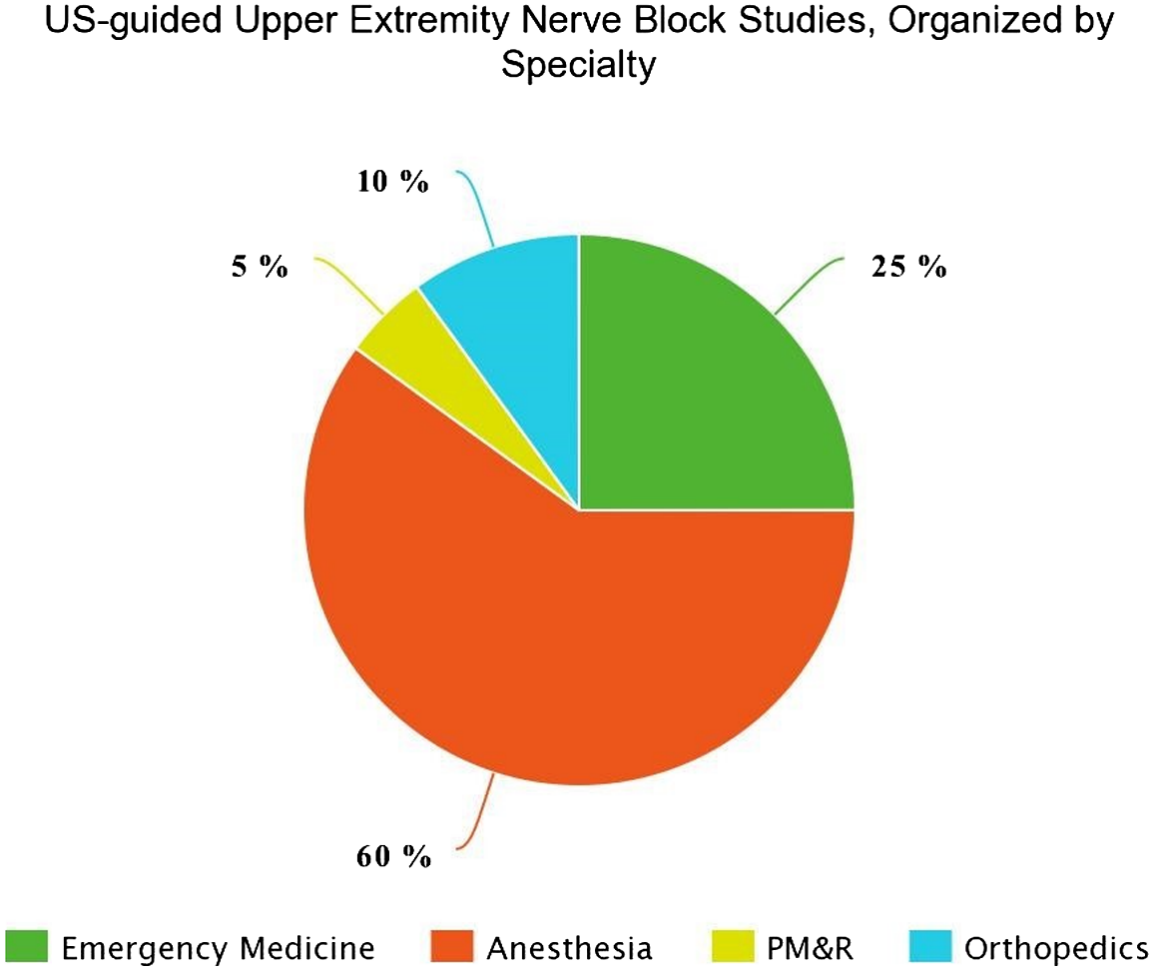
Total proportion of studies included in qualitative analysis by specialty. *US*, ultrasound; *PM&R*, physical medicine and rehabilitation.

**Table 1:** Summary of published studies investigating the efficacy and safety of ultrasound-guided peripheral forearm nerve blocks.

Authors (Year)	Study Design: N enrolled	No. of Blocks Performed	Mean Age in Years (SD) [Range]	Health Status	Setting (Specialty)	Acute or Chronic Pain	Intervention or Exposure	Control	Outcome	Key Findings	Complications (n); type
**A. Mixed forearm block performed (i.e., median, ulnar, and/or radial)**											
Liebmann et al. (2006)	Single-arm interventional; N = 11	22	39* [21–60]	Patients with hand pathology requiring procedural intervention	ED (EM)	Acute	Ultrasound-guided block of the radial, median, and/or ulnar nerves	NA	VAS	Ten of 11 patients had a clinically meaningful reduction in VAS pain score (>13 mm reduction); median reduction in VAS score was 5.0 cm (IQR 3.0, 8.0; *P* = .003)	0
Frenkel et al. (2015)	Single-arm interventional; N = 10	30	11* [9–17]	Pediatric patients with hand injuries requiring procedural intervention	ED (EM)	Acute	Ultrasound-guided blocks of ulnar, median, and radial nerves combined	NA	VAS	Mean VAS decreased from 5.8 to 0.8 (*P* = .04); seven of 10 with complete resolution of pain	0
Soberon et al. (2015)	RCT; N = 60	30	49* [36–58]	Patients undergoing hand surgery	Perioperative (Anesthesia)	Acute	Ultrasound-guided median, ulnar, or radial nerve blocks	Ultrasound-guided brachial plexus block	Block as primary anesthetic	No difference in the number of blocks able to be used as a primary anesthetic between the two groups (97% vs 93% for forearm and brachial groups, respectively)	0
Sohoni et al. (2016)	RCT; N = 12	18	>18	Healthy volunteers	ED (EM)	Acute	Ultrasound-guided forearm block with a saline placebo wrist block	Landmark-based wrist block with an ultrasound-guided saline forearm block	Pinprick sensation	Ultrasound-guided forearm blocks were significantly more likely to block sensation to pinprick compared to anatomic wrist blocks (78% vs 56%, respectively; *P* = 0.032)	0
Sites et al. (2012)	Cohort	536	55	Patients receiving US-guided nerve block 2003–2011	Regional anesthesia (Anesthesia)	Acute	Ultrasound-guided wrist blocks	NA	Postoperative neurologic complications	Only complications reported	0
**B. Median, Ulnar Combined studies**											
Macaire et al. (2008)	RCT; N = 60	30	48 (12)	Patients undergoing carpal tunnel release	Perioperative (Anesthesia)	Acute	Ultrasound-guided median and ulnar nerve blocks at the wrist	Nerve stimulation guided nerve block of median and ulnar nerve	VAS; success rate of block	No difference in VAS scores during post-block venipuncture (30 vs 30, *P* = 0.26). Success rate was 93% in both groups.	1; Transient mechanical paresthesia
**C. Ulnar**											
Marhofer et al. (2013)	RCT; N = 24	12	NR	Healthy volunteers	Research ward (anesthesia)	Acute	Ultrasound-guided ulnar nerve block	Ultrasound-guided ulnar nerve block with added dexmedetomidine	Success rate of block	Complete sensory block in all patients was achieved in both groups.	0
Marhofer et al. (2019)	Crossover study; N = 24	72	30* [22–55]	Healthy volunteers	Research ward (anesthesia)	Acute	Ultrasound-guided ulnar nerve block	Ultrasound-guided ulnar nerve block with added dexamethasone	VAS to pinprick	6.87 (5.78–7.93) hour duration, 6.0 (4.5–10.0) minute onset of ropivacaine block, no relevant effect of dexamethasone on sensory block	0
**D. Median**											
Dufour et al. (2012)	RCT; N = 100	NR	I:40.5 (15.6); C: 41.0 (18.2)	Healthy volunteers	Perioperative (Anesthesia)	Acute	Ultrasound-guided median nerve block	Ultrasound-guided median nerve block with prior dextrose 5% hydrodissection	Cold and light touch block to index finger and thenar eminence	No difference in the percentage of patients with complete cold and light touch blocks at index finger (100% vs 98.1% and 95.2% vs 96.2%) and thenar eminence (100 v 98.1 and 97.5 vs 88.2) between groups	2 in group without D5 hydrodissection; Intraneural injections
Marhofer et al. (2014)	Crossover study; N = 21	42	NR [18–45]	Healthy volunteers	Research ward (anesthesia)	Acute	Ultrasound-guided median nerve block with circumferential spread	Ultrasound-guided median nerve block with non-circumferential spread	Success rate of sensory block	The intended circumferential spread group had a higher success rate compared to non-circumferential spread group (100% v 81%, *P* < 0.05).	0
Liu et al. (2018)	RCT	50	26 months [NR]	Pediatric patients undergoing trigger thumb surgery	Perioperative (Anesthesia)	Acute	Ultrasound-guided median nerve block	Landmark-guided median nerve block	m-CHEOPS*	50/50 successful median nerve blocks in US group, 37/50 of landmark group	0
**E. Radial**											
Unluer et al. (2008)	Single arm interventional	15	NR	Patients with Colles fracture	ED (EM)	Acute	Ultrasound-guided radial nerve block	NR	VAS for fracture reduction	VAS decreased from 8.2 (7.6–8.8) preprocedure to 3.53 (2.73–4.34) postprocedure	0
Okmen et al. (2018)	RCT	25	50 [46–64]	Patients with osteoarthritis	Research ward (PM&R)	Chronic	Ultrasound-guided block superficial branch of radial nerve + exercise	Exercise alone	VAS after 2, 4 weeks	VAS decreased from 8 (6–9) to 1 (0–6) after four weeks for nerve block group, significant difference from exercise group	NR

*NA*, not available; *PM&R*, physical medicine and rehabilitation; *VAS*, visual analog score; *EM*, emergency medicine; *ED*, emergency department; *m-CHEOPS*, modified Children’s Hospital of Eastern Ontario Pain Scale; *NR*, not reported; *RCT*, randomized controlled trial.

* Indicates median age.

**Table 2:** Summary of published studies investigating the efficacy of ultrasound-guided shoulder nerve blocks.

Authors (Year)	Study Design: N enrolled	No. of Blocks Performed	Mean Age in Years (SD) [Range]	Health Status	Setting (Specialty)	Acute or Chronic Pain	Intervention or Exposure	Control	Outcome	Key Findings	Complications (n); type
**A. Suprascapular and Axillary**											
Lee et al. (2014)	RCT; N = 42	21	55.8 (39–72)	Patients with torn rotator cuffs	Perioperative (Anesthesia)	Chronic	Ultrasound-guided suprascapular + axillary nerve block with ropivacaine	Ultrasound-guided suprascapular nerve block alone	VAS	Significant reduction in VAS with suprascapular block + axillary block compared to suprascapular alone	13/21; Rebound pain. No paresthesia or long-term complications
Dhir et al. (2016)	RCT; N = 60	30	46.5 (SD 14.5)	Patients undergoing arthroscopic shoulder surgery	Perioperative (Anesthesia)	Acute	Ultrasound-guided suprascapular + axillary nerve block prior to general anesthesia	Ultrasound-guided interscalene block of brachial plexus	NRS score	Static NRS score of 5.45 in PACU, 4.00 6 hours post-operatively for suprascapular + axillary. Significantly less analgesia than interscalene brachial plexus block 6 hr	0
**B. Suprascapular**											
Tezel et al. (2014)	RCT; N = 41	21	24 (21–73)	Patients with shoulder dislocation	ED (EM)	Acute	Ultrasound-guided suprascapular nerve block	Sedation analgesia with 1–2 mg/kg of ketamine	VAS	VAS decreased from mean 85 (70–98) to mean 45 (33–55). Mean time to discharge significantly lower in nerve block group compared to sedation group	0
Coory et al. (2019)	RCT; N = 42	21	70 (43–85)	Patients with rotator cuff tears	Outpatient (orthopedics)	Chronic	Ultrasound-guided suprascapular nerve block	Subacromial injection	Constant-Murley Score at 12 weeks post-injection	Suprascapular nerve block outperformed Subacromial injection, improved CM score from 35.3 (SD 12.8) to 57.6 (SD 10) in 12 weeks	NR
Ferre et al. (2020)	RCT; N = 83	83	56.6 (11.6)	Patients undergoing shoulder surgery	Perioperative (Anesthesia)	Acute	Ultrasound-guided anterior suprascapular block	Ultrasound-guided posterior suprascapular block	Diaphragmatic excursion on US	41% of anterior (17/42) and 2% of posterior (1/41) had some hemidiaphragmatic paralysis	18/83; Hemidiaphragmatic paralysis of any kind
Bae et al. (2020)	RCT; N = 47	47	55.3 [39–76]	Patients with adhesive capsulitis	Outpatient (orthopedics)	Chronic	Proximal approach to ultrasound-guided suprascapular nerve block	Distal approach to ultrasound-guided suprascapular nerve block	VAS at week 12	Stratified by proximal vs. distal approach: proximal had VAS decrease from 6.8 +/− 1.5 baseline to 3.7 +/− 1.3 at 12 weeks. Distal VAS pain score improved from 6.2 +/− 1.6 to 3.6 +/− 2.0 at 12 weeks.	0
Lim et al. (2020)	RCT; N = 40	40	41.8	Patients undergoing arthroscopic shoulder surgery	Perioperative (Anesthesia)	Acute	Ultrasound-guided anterior and posterior suprascapular nerve block	Ultrasound-guided interscalene block of brachial plexus	Pain Score, FVC reduction	Both anterior suprascapular (19/20) and posterior suprascapular (19/20) had highly effective blockade at 30 minutes, less reduction of FVC compared to interscalene block	Compared SSB to brachial plexus block, less respiratory effect with SSB

NRS scale: 0 = no pain to 10 = worst pain imaginable. Constant Murley Score: combined functional and pain score for shoulder injury (0–100)

*US*, ultrasound; *mg/kg*, milligram/kilogram; *PACU*, post-anesthesia care unit; *FVC*, forced expiratory volume; *SSB*, suprascapular-axillary nerve block, VAS, visual analog score; *EM*, emergency medicine; *ED*, emergency department; *NR*, not reported; *RCT*, randomized controlled trial.
