# Supplementary material for: Block Time: A Multispecialty Systematic Review of Efficacy and Safety of Ultrasound-guided Upper Extremity Nerve Blocks
Source: West J Emerg Med. 2023 Jun 30;24(4):774–85. doi: 10.5811/westjem.56058 (PMC10393451; doi:10.5811/westjem.56058)
Supplement: Supplementary file 1 [file wjem-24-774-s001.docx]

**Appendix 1. Search string for OVID and EMBASE**

**OVID Search String**

**1. exp "anesthesia, conduction"/**

**2. exp "ultrasonography"/**

**3. exp "upper extremity"/**

**4. exp "median nerve"/**

**5. exp "ulnar nerve"/**

**6. exp "radial nerve"/**

**7. exp "musculocutaneous nerve"/**

**8. 3 or 4 or 5 or 6 or 7**

**9. 1 and 2 and 8**

**EMBASE Search String**

**
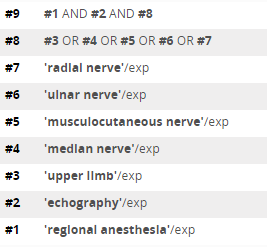
**

**Appendix 2**. **Eligibility criteria for inclusion and exclusion of studies**

| **Category** | **Inclusion Criteria** | **Exclusion Criteria** |
| --- | --- | --- |
| Study Design | *Any clinical study or case study*:   - Randomized controlled trials - Prospective cohort studies - Nested case-controlled studies - Mendelian randomization studies - Non-randomized controlled trials including quasi-experimental and controlled before-and-after studies - Uncontrolled trials - Prospective cohort studies - Retrospective cohort studies - Nested case-controlled studies - Cross-sectional studies - Case-controlled studies | Narrative reviews  Meta-analyses  Systematic reviews  Letters to the editor (as long as they do not include a case study)  Protocols  Background reading  Case reports/case studies |
| Study Duration | No restriction | NA |
| Sample Size | No restriction | NA |
| Intervention/Exposure | Ultrasound-guided regional anesthesia (nerve block) of upper extremity distal nerves (musculocutaneous, ulnar, median, radial, suprascapular, axillary nerves) | Local anesthesia  Regional anesthesia without ultrasound guidance  Regional anesthesia with nerve stimulation guidance  US-guided regional anesthesia to cervical plexus, torso, lower extremities only  Nerve blocks at level of brachial plexus only  Neurolysis or permanent nerve destruction |
| Comparator | Comparisons between different approaches to nerve blocks  Comparisons of nerve blocks to other forms of pain management (local, pharmacologic, general anesthesia)  No comparator  Placebo  No intervention | NA |
| Outcomes | Any outcomes related to pain and pain management  Safety of nerve blocks | Other outcomes including volume of anesthetic, duration to onset, duration of block |
| Date of Publication | No restriction | NA |
| Publication Status | Articles published in peer-reviewed journals | Articles not published in peer-reviewed journals, including unpublished data, manuscript reports, abstracts, pre-prints, and conference proceedings |
| Language of Publication | English | Languages other than English |
| Country | No restriction | NA |
| Study Participants | Human subjects | Non-human subjects  In vitro |

**Appendix 3: Included Studies in Qualitative Analysis, listed alphabetically**

| Authors | Title | Journal | Year |
| --- | --- | --- | --- |
| Bae, K. H., Park, K. C., Jeong, G. M. and Lim, T. K. | Proximal vs Distal Approach of Ultrasound-guided Suprascapular Nerve Block for Patients With Adhesive Capsulitis of the Shoulder: Prospective Randomized Controlled Trial | Archives of Physical Medicine and Rehabilitation | 2020 |
| Coory, J. A., Parr, A. F., Wilkinson, M. P. and Gupta, A. | Efficacy of suprascapular nerve block compared with subacromial injection: a randomized controlled trial in patients with rotator cuff tears | Journal of Shoulder and Elbow Surgery | 2019 |
| Dhir, S., Sondekoppam, R. V., Sharma, R., Ganapathy, S. and Athwal, G. S. | A Comparison of Combined Suprascapular and Axillary Nerve Blocks to Interscalene Nerve Block for Analgesia in Arthroscopic Shoulder Surgery: An Equivalence Study | Regional anesthesia and pain medicine | 2016 |
| Dufour, E., Donat, N., Jaziri, S., Kurdi, O., Couturier, C., Dreyfus, J.-F. and Fischler, M. | Ultrasound-guided perineural circumferential median nerve block with and without prior dextrose 5% hydrodissection: a prospective randomized double-blinded noninferiority trial | Anesthesia and analgesia | 2012 |
| Ferre, F., Pommier, M., Laumonerie, P., Ferrier, A., Menut, R., Bosch, L., Balech, V., Bonnevialle, N. and Minville, V. | Hemidiaphragmatic paralysis following ultrasound-guided anterior vs. posterior suprascapular nerve block: a double-blind, randomised control trial | Anaesthesia | 2020 |
| Frenkel, O., Liebmann, O. and Fischer, J. W. | Ultrasound-guided forearm nerve blocks in kids: a novel method for pain control in the treatment of hand-injured pediatric patients in the emergency department | Pediatric emergency care | 2015 |
| Lee, J. J., Kim, D.-Y., Hwang, J.-T., Lee, S.-S., Hwang, S. M., Kim, G. H. and Jo, Y.-G. | Effect of ultrasonographically guided axillary nerve block combined with suprascapular nerve block in arthroscopic rotator cuff repair: a randomized controlled trial | Arthroscopy : the journal of arthroscopic & related surgery : official publication of the Arthroscopy Association of North America and the International Arthroscopy Association | 2014 |
| Liebmann, O., Price, D., Mills, C., Gardner, R., Wang, R., Wilson, S. and Gray, A. | Feasibility of forearm ultrasonography-guided nerve blocks of the radial, ulnar, and median nerves for hand procedures in the emergency department | Annals of emergency medicine | 2006 |
| Lim, Y. C., Koo, Z. K., Ho, V. W., Chang, S. S., Manohara, S. and Tong, Q. J. | Randomized, controlled trial comparing respiratory and analgesic effects of interscalene, anterior suprascapular, and posterior suprascapular nerve blocks for arthroscopic shoulder surgery | Korean Journal of Anesthesiology | 2020 |
| Liu, W., Liu, J., Tan, X. and Wang, S. | Ultrasound-guided lower forearm median nerve block in open surgery for trigger thumb in 1- to 3-year-old children: A randomized trial | Paediatric anaesthesia | 2018 |
| Macaire, P., Singelyn, F., Narchi, P. and Paqueron, X. | Ultrasound- or nerve stimulation-guided wrist blocks for carpal tunnel release: a randomized prospective comparative study | Regional anesthesia and pain medicine | 2008 |
| Marhofer, P., Columb, M., Hopkins, P. M., Greher, M., Marhofer, D., Bienzle, M. and Zeitlinger, M. | Dexamethasone as an adjuvant for peripheral nerve blockade: a randomised, triple-blinded crossover study in volunteers | British journal of anaesthesia | 2019 |
| Marhofer, D., Karmakar, M. K., Marhofer, P., Kettner, S. C., Weber, M. and Zeitlinger, M. | Does circumferential spread of local anaesthetic improve the success of peripheral nerve block? | British journal of anaesthesia | 2014 |
| Marhofer, D., Kettner, S. C., Marhofer, P., Pils, S., Weber, M. and Zeitlinger, M. | Dexmedetomidine as an adjuvant to ropivacaine prolongs peripheral nerve block: a volunteer study | British journal of anaesthesia | 2013 |
| Metin Ökmen, B., Ökmen, K. and Altan, L. | Effectiveness of superficial radial nerve block on pain, function and quality of life in patients with hand osteoarthritis: A prospective, randomized and controlled single-blind study | Archives of Rheumatology | 2018 |
| Sites BD, Taenzer AH, Herrick MD, Gilloon C, Antonakakis J, Richins J, Beach ML. | Incidence of local anesthetic systemic toxicity and postoperative neurologic symptoms associated with 12,668 ultrasound-guided nerve blocks: an analysis from a prospective clinical registry. | Regional Anesthesia and Pain Medicine | 2012 |
| Soberon, J. R., Jr., Crookshank, J. W., 3rd, Nossaman, B. D., Elliott, C. E., Sisco-Wise, L. E. and Duncan, S. F. | Distal Peripheral Nerve Blocks in the Forearm as an Alternative to Proximal Brachial Plexus Blockade in Patients Undergoing Hand Surgery: A Prospective and Randomized Pilot Study | The Journal of hand surgery | 2016 |
| Sohoni, A., Nagdev, A., Takhar, S. and Stone, M. | Forearm ultrasound-guided nerve blocks vs landmark-based wrist blocks for hand anesthesia in healthy volunteers | The American journal of emergency medicine | 2016 |
| Tezel, O., Kaldirim, U., Bilgic, S., Deniz, S., Eyi, Y. E., Ozyurek, S., Durusu, M. and Tezel, N. | A comparison of suprascapular nerve block and procedural sedation analgesia in shoulder dislocation reduction | The American journal of emergency medicine | 2014 |
| Unluer, E. E., Karagoz, A., Unluer, S., Kosargelir, M., Kizilkaya, M., Alimoglu, O., Akoglu, H. and Aslan, C. | Ultrasound-guided supracondylar radial nerve block for Colles Fractures in the ED | The American journal of emergency medicine | 2016 |

**Appendix 4. Results of Risk-of-Bias Assessment of all included randomized control and cross-over studies.**

| **Risk-of-Bias Assessment** | | | | | | | |
| --- | --- | --- | --- | --- | --- | --- | --- |
| Author, Year | Outcome | Study Design | Domain 1 | Domain 2 | Domain 3 | Domain 4 | Domain 5 |
| Soberon, 2015 | Block as primary aesthetic | RCT | Low | Low | Low | Some | Some |
| Sohoni, 2016 | Pinprick sensation | RCT | High | Low | Low | Low | Some |
| Macaire, 2008 | VAS | RCT | Low | High | Low | Low | Some |
| Marhofer, 2013 | Success rate | RCT | Some | Low | Low | Low | Some |
| Marhofer, 2019 | VAS to pinprick | Cross-over | Low | Low | Low | Low | Low |
| Dufour, 2012 | Cold and light touch sensation | RCT | Low | High | Low | Some | Some |
| Marhofer, 2014 | Success rate | Cross-over | Low | Some | Low | Low | Some |
| Liu, 2018 | m-CHEOPS | RCT | Low | High | Low | Some | Some |
| Okmen, 2018 | VAS | RCT | Some | Some | Low | Low | Some |
| Lee, 2014 | VAS | RCT | Low | Low | Low | Low | Some |
| Dhir, 2016 | Pain score | RCT | Low | Low | Low | Low | Low |
| Tezel, 2014 | VAS | RCT | Low | Low | Low | Some | Some |
| Coory, 2019 | Constant Murley scores | RCT | Low | Low | Low | Low | Some |
| Ferre, 2020 | Hemidiaphragmatic paralysis | RCT | Low | Low | Low | Low | Some |
| Bae, 2020 | VAS | RCT | Low | Low | Low | Low | Low |
| Lim, 2020 | Sensation in suprascapular | RCT | Low | Low | Low | Low | Low |
| Lim, 2020 | Reduction in FVC | RCT | Low | Low | Low | Low | Low |

Domain 1: Risk of bias arising from the randomization process

Domain 2: Risk of bias due to deviations from the intended interventions

Domain 3: Risk of bias due to missing outcome data

Domain 4: Risk of bias in measurement of the outcome

Domain 5: Risk of bias in selection of the reported result
